# Pathogenic variants of *PROC* gene caused type I activity deficiency in a familial Chinese venous thrombosis

**DOI:** 10.1111/jcmm.14563

**Published:** 2019-07-23

**Authors:** Yongjian Yue, Shengguo Liu, Xuemei Han, Lu Xiao, Qijun Huang, Shulin Li, Kaixue Zhuang, Mo Yang, Chang Zou, Yingyun Fu

**Affiliations:** ^1^ Key Laboratory of Shenzhen Respiratory Diseases, Department of Pulmonary and Critical Care Medicine, Institute of Shenzhen Respiratory Diseases, The First Affiliated Hospital of Southern University of Science and Technology, The Second Clinical Medical College of Jinan University Shenzhen People's Hospital Shenzhen Guangdong China; ^2^ The Seventh Affiliated Hospital of Sun Yat‐sen University Shenzhen Guangdong China; ^3^ Clinical Medical Research Center, The First Affiliated Hospital of Southern University of Science and Technology, The Second Clinical Medical College of Jinan University Shenzhen People's Hospital Shenzhen Guangdong China

**Keywords:** p.Ala178Pro, PROC deficiency, rs199469469, venous thromboembolism, whole exome sequencing

## Abstract

Pathogenic mutation of protein C (*PROC*) gene results into the deficiency of PROC activity. This study aimed to identify the pathogenic genetic variants and to explore the functional consequence in Chinese familial venous thrombosis (VTE). Whole exome sequencing was performed to identify the pathogenic variants of anticoagulant factors. Serum coagulation and anti‐coagulation factors activity were assayed to evaluate the genetic association. Functional study of PROC antigen secretion deficiency was conducted in VTE subjects and in vitro cell lines. One rare pathogenic variant (p.Ala178Pro) was identified in the four VTE subjects but not in the normal subjects from the family. An inframeshift variant (rs199469469) was also identified in a paediatric subject of the pedigree. Further evaluation of serum PROC activity levels in p.Ala178Pro variants VTE carriers showed significantly lower PROC activity compared to non‐carriers. Furthermore, in vitro study showed that the p.Ala178Pro mutant cells had a consistent reduction in concentration of PROC antigen. In conclusions, our study demonstrated the pathogenic variant (p.Ala178Pro) contributed to PROC type I activity deficiency, which may be due to decreased secretion of PROC.

## 1 INTRODUCTION

Venous thromboembolism (VTE) is a complex and multi‐factorial thrombotic disorder that includes deep venous thrombosis (DVT), cerebral infarction and pulmonary embolism (PTE). The hospitalization rates of VTE increased from 3.2 to 17.5 per 100 000 population, and the mortality decreased from 4.7% to 2.1% in China.^1^ The acquired or inherited risk factors for VTE development include surgery, pregnancy, cancer. The genetic factors account for up to 60% of the variation in susceptibility to VTE.^2^

Mutations in the genes of coagulation system caused deficiencies of antithrombin (AT), protein C (PROC), protein S (PROS1) and other coagulation factors, which are major contributors for the susceptibility to VTE.^3^ PROC is a vitamin K‐dependent serine protease zymogen in plasma, and activated protein C (APC) exerts its anticoagulant function through inactivation of the blood coagulation factors FVa and FVIIIa.^4^ Based on the functional and immunological PROC assays, PROC deficiency can be divided into two types. Type I deficiency is characterized by a reduction in the antigen concentration and activity function, whereas type II deficiency is featured by normal or increased antigen concentration but reduced activity.^5^ Homozygous or compound heterozygous PROC deficiency usually leads to VTE. Previous studies already identified hundreds of *PROC* and *PROS1* mutations; however, the mutation pattern is different among Western and Asian populations.^6^ These variants in the individuals only partly contributed to the PROC activity deficiencies, suggesting other pathogenic mutations may be involved.^7^

The aim of the study is to investigate whether familial VTE patients with low APC level would carry PROC or PROS1 genetic variants and to evaluate the association among VTE and PROC or PROS1 deficiencies in the Chinese population. Identification of hereditary thrombophilic genetic factors would enable prognosis, prevention and genetic counselling for VTE patients and family members.

## 2 MATERIALS AND METHODS

### 2.1 Subjects

A total of 212 sporadic VTE patients and one family with four VTE patients and three normal siblings were recruited during December 2013 to June 2018 in the Shenzhen People's Hospital.^8^ The PTE patients were diagnosed according to the criteria released by European Society of Cardiology (ESC) in 2014.^9^ In addition, a total of 196 sporadic control subjects were recruited. The recruited criterions of healthy controls were same as we described before.^10^ Informed consent was obtained from all the patients and healthy individuals before enrolled in the study. All the research procedures are approved by the Ethics Committee of Shenzhen People's Hospital. All procedures performed in studies involving human participants were in accordance with the 1964 Declaration of Helsinki ethical standards.

### 2.2 Whole exome sequencing and variants validation

Qualified genomic DNA was extracted from the whole blood, and capture‐based target enrichment was performed by Agilent SureSelect All Exon V5 capture kit according to the manufacturer's protocols. Massively parallel sequencing was performed by HiSeq4000 platform (Illumina). All variants including nonsynonymous, insertion or deletion and non‐coding variants occurring in the genomic region among *PROC* were evaluated. The variants with MAF and function were annotated by databases of gnomeAD, avsnp150, and so on. Tolerant (SIFT), Polyphen2 HumVar and CADD (combined annotation dependent depletion) were used to assess the pathogenesis of these variants. Sanger sequencing was performed to validate all candidate variants in the VTE familial cohort.

### 2.3 Serum coagulation and anti‐coagulation factors activity assays

Blood samples of VTE were collected into Na‐citrate containing vacutainer tube. Plasma was obtained by centrifugation at 2000 *g* for 15 minutes at 4°C and stored at −80°C until use. The PROC activity was determined by the automatic chromogenic assay on the IL coagulation systems (Instrumentation Laboratory) according to the manufacturers' instructions. The susceptible thrombosis factors were also detected in the proband and siblings including PROS1, antithrombin anticoagulant activity, fibrin degradation product (FDP), plasma fibrinolytic enzyme activity assay (PLGA) and lupus‐like anticoagulant (LlA).

### 2.4 Cell culture

The effect of p.Ala178Pro PROC antigen secretion and synthesis was analysed by in vitro functional studies in HEK‐293T and ECV‐304 cells. The mutagenesis of PROC mutants (c.532G>C) was performed by high‐fidelity PCR using the appropriate primers (F: ACCTCCTGCAGTGTCACCCCCCAGTGAAGTTCCCTTGTGGG, R: CCCACAAGGGAACTTCACTGGGGGGTGACACTGCAGGAGGT). The plasmid was re‐constructed based on the plasmid of pLVX‐Puro. Mutant plasmid (plvx‐CMV‐MCS‐EF1alpha‐EGFP‐PGK‐puro) was confirmed by sequencing. Empty control, wild‐type and mutant PROC protein plasmids were labelled with green fluorescent (GFP) for normalizing transfection efficiency. For HEK‐293T cells, transient co‐transfection was conducted using Lipofectamine 2000 reagent (Invitrogen). PROC protein plasmids and PMD2.G, pMDLg‐pRRE, pRSV‐Rev were co‐transfected into HEK‐293T cells by Lipofectamine 2000 reagent to package lentivirus. Lentivirus was used for ECV‐304 cell stable transfection.

### 2.5 In vitro functional assays

The cell lysates and culture conditioned media were harvested at 72 hours after transfection. Briefly, nine well culture media were mixed and then freeze concentrated for improving the PROC antigen since the concentration is too low to be detectable. The PROC antigen and expression levels in cell lysates were normalized against the transfection efficiency. The secreted PROC antigen concentrations in culture media were measured by ELISA kit (Abnova) according to the manufacturer's protocol.

### 2.6 Statistical analysis

SPSS 13.0 (IBM) was used for statistical analysis. Independent‐samples *t* test was conducted among the case‐control groups. The results with *P* value less than .05 were considered statistically significant.

## 3 RESULTS

### 3.1 Patient characteristics

**Figure 1:**
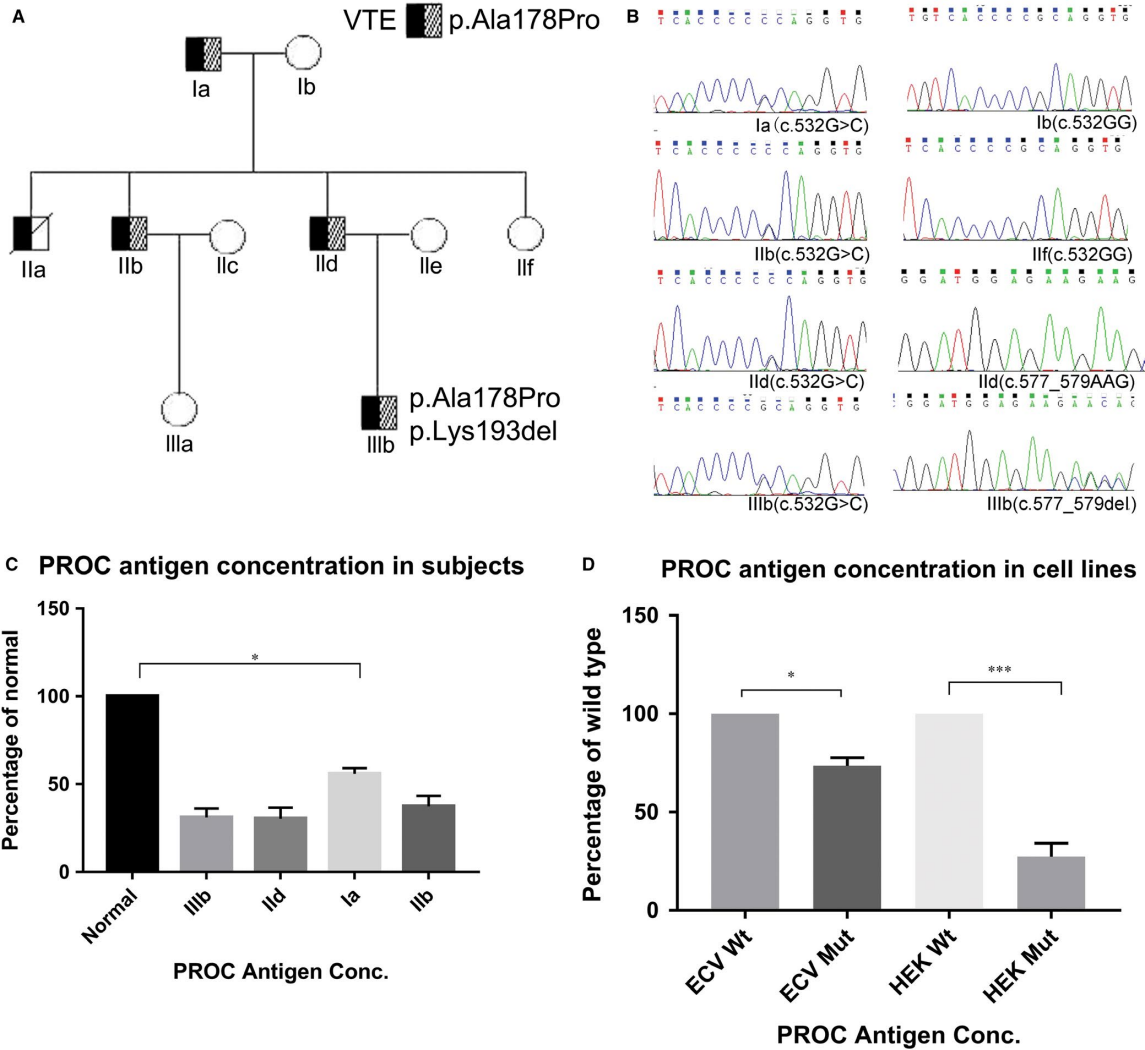
A, The pedigree of the 3‐generation Chinese family. The proband (IId) carried heterozygous mutations: c.532G>C, inherited from his father (Ia). IIIb carried two coding mutations (c.532G>C and c.577_579del, p.Lys193del). Black symbols indicate VTE subjects and hatched squares indicate c.532G>C carriers. B, Sanger validation results of the two variants. All VTE subjects carried c.532G>C mutation but not the normal familial members of IIf and Ib (part A). Only IIIb carried the mutation of c.577_579del. C, The secretion of PROC antigen in VTE familial subjects was detected by Elisa. D, In vitro secretion concentration of PROC antigen in two cell lines. (wt: wild type, mut: mutant)

In the pedigree of the 3‐generation Chinese family, the proband showed phenotype of PTE with very low PROC activity. Among the family members, three subjects had PTE (IIa, IIb and IId), one had cerebral infarction (Ia) and one had deep vein thrombosis and PTE (IIIb). The patient of IIa died of PTE. Three normal familial members (Ib, IIe, IIf) did not showed any clinical thrombotic phenotype (Figure 1A).

### 3.2 Pathogenic variants were identified by whole exome sequencing

Whole exome sequencing was conducted in the family subjects to identify the inherited genetic factors. All coding variants presented in the coding region among *PROC* and *PROS1* were identified. The variants with MAF geater than 5% in databases (gnomeAD) were considered as common variants. After filtering within‐house bioinformatics pipeline, 11 variants were identified in PROC and only two variants (NM_000312: p.A178P, p.K193del) are located in exon region with rare frequency (Table S1). The variant allele minor frequency (MAF) of p.Ala178Pro and p.Lys193del is 0.00059% and 0.011% in the East‐Asian population (gnomeAD), respectively. Five VTE subjects carried the heterozygote PROC polymorphism (c.532G>C, p.Ala178Pro) which were not presented in controls (Figure 1B). Further case‐control genotyping study demonstrated that the two variants were not presented in 196 normal and 212 sporadic VTE subjects. Pathogenesis evolution results of CADD showed the PHRED value is 13, which indicates that these are predicted to be the 10% most deleterious substitution. In the family members, only two subjects (IIb and IId) showed protein S deficiency, while we did not find any coding variant in *PROS1* and PROCR (PROC receptor) among the VTE cases (Table 1). The functional deficiency consequences of PROS1 might be caused by the regulatory effects of other unknown genetic changes. FDP and PLGA were found to be within normal ranges in all the patients.

**Table 1:** Coagulation, anti‐coagulation factors activity levels, and *PROC* coding variants in all the family members

Subjects		APC (%)	PROS1 (%)	AT III (%)	FDP (μg/mL)	PLGA (%)	LlA (%)	*PROC* coding variants
	Normal range	65‐140	63.5‐149	82‐128	0‐2.01	80.2‐132.5	Negative	
Ia	VTE	48	117.8	87	0.7	83	Negative	c.G532C
Ib	Normal	106	83.1	104	1.45	95	Negative	–
IIb	PTE	23	31.3	114	0.35	103	Positive	c.G532C
IId	PTE	25	51.5	91	0.31	85	Negative	c.G532C
IIe	Normal	116	74.1	92	0.75	99	Negative	–
IIf	Normal	135	113.9	116	0.79	115	Negative	–
IIIb	VTE	43	77.9	82	0.43	88	Positive	c.G532C, c.577‐579del

### 3.3 PROC activity deficiency analysis in variants carrying subjects

The activities of serum coagulation and anti‐coagulation factors were measured to check the association between genetic defects and clinical phenotype. The normal range of PROC and antithrombin anticoagulant activity levels is based on the values derived from thousands of subjects in clinical diagnosis. The normal range of PROS1 was performed using the protein S activity kit on representative members of the ACL TOP family systems. All normal family members showed normal PROC activity level. The activity percentage of serum APC level is much lower in VTE subjects compared to all normal siblings. Serum antithrombin activity and PLGA level are normal in all the family members, while IIIb was LIA‐positive (Table 1).

### 3.4 Effect of p.Ala178Pro mutation on PROC antigen secretion and synthesis

To characterize the influence of the p.Ala178Pro mutation on PROC antigen secretion and synthesis, the PROC secretion in VTE subjects and in vitro cells was detected. The intracellular protein expression of PROC is similar in each VTE individual. The ELISA results showed secretion of serum PROC was reduced by 31.8%‐55% in the VTE subjects compared with control (Figure 1C). The mean concentration of PROC antigen decreased to 58% in VTE subjects. Interestingly, the serum PROC antigen secretion reduction is consistent with the reduction of APC, such as IIb and IId showed the lowest serum PROC secretion and APC. In vitro study demonstrated that the mRNA expression and intracellular protein expression of PROC were not significantly different between wild‐type and mutant cell lysates. However, PROC secretion in conditioned media from mutant cells reduced by 25% in ECV‐304 and by 68% in HEK293T cells, respectively, compared with wild‐type cells (Figure 1D).

## 4 DISCUSSION

In this study, we identified a rare pathogenic *PROC* variant (p.Ala178Pro) in a Chinese family with VTE by whole exome sequencing. Two coding variants were presented in the PROC activity deficiency family. The identified variant (p.Ala178Pro) cosegregated with PROC activity deficiency in the VTE family and was not identified in the 196 normal and 212 sporadic VTE subjects. Our in vitro study confirmed that the p.Ala178Pro mutation can lead to PROC activity deficiency which may be due to abnormal secretion of PROC.

The three‐dimensional structure of PROC comprise the Gla domain, two epidermal growth factor (EGF)‐like regions, an activation peptide and the enzymatic serine protease domain.^4^ APC is generated after proteolysis between Arg211 and Leu212 (UniProt:P04070) by thrombin‐throm‐bomodulin complex, which is a trypsin‐like serine protease with a typical serine protease active site triad (253His, 299Asp, 402Ser) in the endothelial cells. Previous studies showed that the mutations in the Gla or serine proteinase domain of PROC can results in type I deficiency.^11^ The p.Ala178Pro variant was not located in serine proteinase domain but it resided adjacent to the autolysis loop (184Gly‐195Ser) and located the vitamin K‐dependent PROC light chain. The identified *PROC* (p.Ala178Pro) variant cosegregated with PROC activity deficiency but this cosegregation could also arise by chance considering the low MAF of the variant in the South‐East population. Previous studies showed the reduced anticoagulant activity of PROC was associated with other two nearby mutations of p.Arg189Trp and p.Lys193del in the Chinese population.^12^ The genetic mutations of p.Ala178Pro that would impair PROC folding or degradation in endoplasmic reticulum or Golgi apparatus which may lead to the APC generation decreased.

To further elucidate the association between genetic mutations and PROC deficiency, the protein intracellular expression and extracellular secretion were examined in wild‐type and mutant cells. Previous in vitro study showed that most of missense mutations of PROC resulted in a decreased secretion of PROC, which was resulted from aberrant protein structure or changes in substrate recognition sites.^13^ Our in vitro expression analysis revealed that the variant p.Ala178Pro mainly impaired the extracellular secretion of PROC but had no effect on the intracellular expression of PROC. In vitro results were consistent with the phenotype of VTE mutation carriers. That indicated the variant p.Ala178Pro is high possibility caused the type I PC deficiency. Further functional studies are required to elucidate the molecular mechanism regarding the deleterious effects of p.Ala178Pro mutation on PROC activity deficiency.

Our study identified a novel coding variant of rs199469469 (p.Lys193del) that only presented in the young DVT patient IIIb. Previous study showed that rs199469469 was a common variant and was significantly associated with high risk of venous thrombosis with OR greater than 2.8.^7^, ^14^ Linear regression results showed that the PROC rs199469469 variant had the most significant correlation with the anticoagulant activity level of PROC, but some carriers was still within the normal range anticoagulant activities.^14^ Other studies in Asian population also showed rs199469469 exist in normal groups but showed decreased protein C activity and significant association with PTE risk.^15^ The prevalence of PROC rs199469469 was approximately 6.78% in VTE patients and 2.42% in healthy controls of the Chinese population.^14^ The reference database of gnomeAD showed this variant only exist in East Asia population with frequency less than 1%. The population attributable risk of rs199469469 is 4.14% in Chinese population.^7^ However, whether the common variant rs199469469 contributed to venous thrombosis and protein C activity deficiency in the Asian population still requires further validation. Several studies suggested that the percentage of abnormal PROC and PROS1 activity in Asian individuals is different from Caucasians.^12^, ^16^ The venous thrombosis may be more likely caused by the coexistence of different genetic variants.

In conclusion, we identified a rare PROC pathogenic variant p.Ala178Pro and this variant was found to be cosegregated with PROC type I deficiency in a familial VTE patients. Our study showed the genetic risk factors would be unique differences among Chinese, Caucasian and other populations. Further studies are required to further elucidate the molecular mechanism regarding the effects of p.Ala178Pro mutation on PROC activity deficiency.

## CONFLICT OF INTEREST

The authors confirm that there are no conflicts of interest.

## AUTHORS' CONTRIBUTIONS

YY and YF prepared the project proposal and study design, supervised the genetic analysis. YY performed analysed all the sequencing data and conducted the statistical analysis. LX, QH and SL conducted sample collection. KZ and XH participated in clinical data interpretation. SL and LX conducted PROC activity detection and in vitro experiment. CZ and MY assisted with the prepared the manuscript and revised the manuscript.

## Data Availability

Data available on request due to privacy/ethical restrictions.
